# Special Issue “Coronary Artery Disease Interventions”

**DOI:** 10.3390/jcm13030817

**Published:** 2024-01-31

**Authors:** Lukas Herold, Gabor G. Toth, Dirk von Lewinski

**Affiliations:** Department of Cardiology, Medical University of Graz, 8036 Graz, Austria; lukas.herold95@gmail.com (L.H.); gabor.g.toth@medunigraz.at (G.G.T.)

The treatment and burden of patients with severe ischemic heart disease, whether acute or chronic, remain some of the greatest challenges in cardiology [1]. Accordingly, this Special Issue entitled “Coronary Artery Disease Interventions” aims to shed light on recent advances in both the interventional and pharmacological strategies of this patient subgroup. It also aims to discuss the collaborative therapies delivered by the disciplines of interventional cardiology, intensive care medicine, and aftercare, based on various research articles and reviews of significant clinical and scientific value.

Herein, we give an overview of relevant research to provide solid scientific evidence.

In the era of expanding possibilities for coronary and non-coronary percutaneous interventions, the site of arterial access has been in focus for decades. As the most common source of periprocedural complications, emphasis has been placed on achieving fundamental improvement, through which radial access has become the default option due to its superiority to femoral access [2,3]. However, further refinement of the technique is inevitable, resulting in the implementation of novel techniques such as distal radial access (dRA), which has gained importance as demonstrated by the growing number of studies that describe several of its advantages [4]. Achim et al. stated the excellent feasibility, safety, and fast learning curve of dRA in their multicenter trial [5]. Furthermore, this access shows promising results through the successful recanalization of radial artery occlusion (RAO) in an additional single-center approach highlighted in this Special Issue [6].

In addition to puncture techniques, starting with bare metal stents, the march of progress did not stop with the development of novel stents either [7]. Despite some of the limitations still present, promising long-term results following the implantation of Magmaris (Biotronik, Berlin, Germany), a second-generation drug-eluting bioresorbable metallic scaffold, in patients with complex coronary lesions might open the door for further breakthroughs in development, as stated by the authors of this article [8].

Bifurcation PCI, which represents up to 30% of all lesions, remains one of the major challenges in coronary interventions. Despite the use of latest-generation drug-eluting stents (DESs), the outcomes after PCI are still inferior compared to non-bifurcation lesions [9,10]. Therefore, finding the optimal stenting technique is one of the “holy grails” of coronary interventions. Evaluating different stenting strategies based on data of a multicenter registry with over 2000 patients, Lim et al. proposed a single-stent strategy in non-LAD bifurcation lesions [11].

Besides complex coronary lesions, Lei et al. highlighted the topic of coronary calcification as the Achilles’ heel of PCI. Here, cases were divided according to different entities of calcified plaques in order to measure their effect on outcomes in a cohort of acute coronary syndrome (ACS) patients. In this context, the occurrence of major adverse cardiac events (MACEs) could be detected more frequently in ACS patients with eruptive calcified nodules, while these kinds of plaque formations also displayed an independent risk factor for MACEs [12].

This Special Issue also discusses the effect of complex percutaneous coronary interventions (PCIs) on ischemic and bleeding events in patients with acute myocardial infarction (AMI). This work highlights that procedure complexity can be identified as an independent risk factor for worse outcome during long-term follow-up periods. As a result, individual decision making and the need for large-volume tests regarding antithrombotic therapy, especially in the setting of complex coronary interventions, have been discussed [13].

On the same line, Lhermusier et al. concentrated on possible differences in the effects of P2Y12 inhibitors after rotational atherectomy (RA) in the TIRATROP study. Although GP IIb/IIIa blockade is known to be associated with a reduction in post-PCI myocardial necrosis, the present study failed to demonstrate the superiority of ticagrelor to clopidogrel in myocardial necrosis measured by troponin enhancement [14,15,16].

In their article about the use of extracorporeal membrane oxygenation (ECMO) in patients with AMI-associated cardiogenic shock, von Lewinski et al. aimed to add real-world experience that would complement randomized-trial-derived data [17]. By applying the ECLS-Shock inclusion criteria on an all-comer cohort of cardiogenic shock (CS) patients from a large CS registry, it was proposed that mechanical circulatory support (MCS) systems might be beneficial in selected real-world cohorts [18,19].

Relevant to the debate about MCS use that has flared up after the release of the ECLS-Shock results, Cankar et al. provided evidence for a favorable long-term outcome of ECMO survivors once discharged from the hospital. This study comprised the longest follow-up of non-surgical ECMO patients with regard to quality of life so far and showed no significant differences between ECMO survivors and the general population to that effect, by using the EQ-5D-5L questionnaire [20].

As a highlight of this Special Issue, two excellent reviews on coronary microcirculation and the current knowledge on coronary no-reflow after PCI address past, current, and future challenges in the world of coronary artery disease [1,21]. These reviews should serve as a motivational breeding ground to enhance ongoing research aiming to reduce the burden of coronary artery disease.

As Guest Editors of this Special Issue, we are delighted to thank the reviewers for their insightful comments, all authors for their valuable contributions, and the JCM staff for their collective support and assistance throughout this process.

## Abbreviations

ACS	acute coronary syndrome
CS	cardiogenic shock
DES	drug-eluting stent
dRA	distal radial access
MACE	major adverse cardiac event
MCS	mechanical circulatory support
PCI	percutaneous coronary intervention
RA	rotational atherectomy
RAO	radial artery occlusion
